# The Reliability of the Timed Up and Go Test among Portuguese Elderly

**DOI:** 10.3390/healthcare11070928

**Published:** 2023-03-23

**Authors:** Filipe Rodrigues, José E. Teixeira, Pedro Forte

**Affiliations:** 1 ESECS—Polytechnic of Leiria, 2411-901 Leiria, Portugal; 2 Life Quality Research Center, 2040-413 Leiria, Portugal; 3 Department of Sport Sciences, Polytechnic of Guarda, 6300-559 Guarda, Portugal; 4 Department of Sport Sciences, Polytechnic of Bragança, 5300-253 Bragança, Portugal; 5 Research Centre in Sports Sciences, Health, and Human Development, 6201-001 Covilhã, Portugal; 6 CI-ISCE ISCE Douro, Higher Institute of Educational Sciences of the Douro, 4560-708 Penafiel, Portugal

**Keywords:** older adult, dynamic balance, reliability, measurement

## Abstract

Assessment of dynamic balance is typically completed through functional tests, such as the Timed Up and Go (TUG) test, which measures the time it takes for an individual to stand up from a chair, walk a set distance, turn around, and sit back down. This test has been validated in several countries. However, in the Portuguese population there is a gap on testing the reliability of this functional test in a sample of the elderly both living in the community or in nursing homes. Thus, this study aimed at examining the reliability of the TUG in a sample of Portuguese elderly. An Intraclass Correlation Coefficient (ICC) analysis was performed between the first time (T1) and the time score after 16 weeks (T2) in TUG test by 38 males and 79 females aged between 60 and 92 years. The results showed acceptable scores of ICC in community-dwelling and nursing home resident elderly in both moments. In addition, significant differences were found between these groups of older adults, showing that community-dwelling elderly show greater agility and balance capacity compared to those living in nursing homes. Thus, the TUG test can be applied in the Portuguese elderly in both community-dwelling and nursing home resident elderly.

## 1. Introduction

As the general population continues to age, the preservation of mobility and balance becomes increasingly crucial in sustaining an independent and active lifestyle. Dynamic balance is an important component of physical health in older persons. However, aging is related with a decline in dynamic balance, which can increase the risk of falls and have a detrimental influence on quality of life. Falls can have long-term physical and psychological repercussions, such as disability, fear of falling, and reduced quality of life [1].

The ability of an individual to maintain stability and control while in motion is referred to as dynamic balance [2]. To establish a consistent and efficient movement pattern, sensory input, motor output, and cognitive processes must be coordinated. Dynamic balance is required for daily activities such as walking, ascending stairs, and getting out of a chair, as well as more sophisticated movements such as picking up an object, turning around, and placing it in an elevated area [3]. Multiple physiological systems must collaborate to establish dynamic balance. Through sensory receptors in the skin, muscles, and joints, the somatosensory system gives information about the body’s position and movement [4]. The visual system offers input about the environment and helps to adapt motions depending on visual cues. The vestibular system in the inner ear gives information about head movement and direction and plays a critical role in maintaining balance during dynamic movements. Motor output is also critical for achieving dynamic balance. The musculoskeletal system must generate the appropriate force and torque to control movements and maintain postural stability [5]. This requires the coordinated activation of multiple muscle groups, as well as the ability to adjust movements based on feedback from sensory systems. Cognitive processes also play an important role in dynamic balance. Attention, memory, and decision-making are all involved in the planning and execution of movements. The ability to anticipate and respond to environmental cues is essential for successful dynamic balance [6]. Age-related declines in sensory, motor, and cognitive function can all contribute to a loss of dynamic balance, and interventions to improve these functions are essential for maintaining independence and quality of life in older adults [7].

### 1.1. Assessing Dynamic Balance in the Elderly

Measuring dynamic balance in the older adult population is important for several reasons [8]. First, dynamic balance is an important component of physical function because it helps people to maintain postural stability while walking, turning, and reaching. Impaired dynamic balance can increase the likelihood of falls, which are a leading cause of injury and loss of independence in the elderly. Second, evaluating dynamic balance can assist healthcare providers in tracking changes in physical function over time as well as monitoring the efficacy of programs aimed at improving mobility and balance in older persons. Finally, evaluating dynamic balance in research settings can be a useful tool for identifying risk factors and potential interventions to improve mobility and balance in the aged population [9].

As the population ages, functional assessment techniques are being utilized more frequently to examine the physical performance of older persons. The Timed Up and Go (TUG) test is a functional evaluation instrument described in the literature by Podsiadlo and Richardson in 1991 [10]. The TUG test has received considerable acceptance as a simple, low-cost, and reliable assessment of functional mobility in older persons since its initial publication [8,11]. The test requires an individual to stand up from a chair, walk 2.44 m, turn around, walk back to the chair, and sit down again, with the time taken to complete the test being recorded. The TUG test has been proved to be a valid and reliable assessment of geriatric mobility and fall risk, and it has been widely utilized in clinical and research contexts. It is specifically regarded as a trustworthy and valid instrument for assessing mobility and fall risk in the elderly around the world.

### 1.2. Balance in Community-Dwelling and Nursing Home Resident Older Adults

There are disparities in dynamic balance between aged people living in the community and those residing in nursing homes, according to extant literature [12]. Nursing home residents frequently have a higher frequency of chronic health issues and functional impairments than community residents. Mobility difficulties, sensory abnormalities, and cognitive decline can all lead to a loss of dynamic balance. Furthermore, nursing home patients may have limited access to exercise and physical activity programs, exacerbating deficits in balance and mobility. Several studies have looked at how dynamic balance differs between community-dwelling older persons and nursing facility residents. One study, for example, discovered that nursing home residents performed much worse on balance than community-dwelling elderly persons [13]. Another study discovered that nursing home patients had considerably lower balance levels than community-dwelling elderly people, indicating poorer dynamic balance [13,14].

### 1.3. Current Research

Functional test reliability assessments are significant because they provide valuable information on the consistency and reproducibility of test outcomes. The extent to which a test delivers consistent and stable results over time and across testing conditions is referred to as reliability in the context of functional evaluations in the older adult population. Reliability assessments can assist healthcare practitioners and researchers in determining if a test is appropriate for clinical or research purposes, as well as providing insight into the precision and accuracy of test results. Reliable function tests are critical in clinical settings for accurate diagnosis, treatment planning, and monitoring of therapy outcomes. Inconsistent results from an unreliable test might lead to false diagnoses, improper treatment decisions, and a loss of faith in the test results. Reliability analyses are vital in research settings to ensure that study outcomes are accurate and meaningful. If a test is untrustworthy, study results may be difficult to interpret or may not correctly reflect the true impact of an intervention.

There is no scientific evidence to support the reliability of the TUG test in the Portuguese elderly. While it has been used in a number of experimental trials [15,16], comprehensive reliability evaluations are still needed to offer evidence of consistency over time in a sample of Portuguese elderly. Reliability is an important component of validity, which refers to the extent to which a test measures what it is intended to measure. By demonstrating that the TUG test is reliable, the current study can provide evidence that the test is a valid and useful tool for assessing functional mobility in Portuguese older adults living in the community or in nursing homes. Furthermore, there has been no research comparing the findings of the TUG test between elderly people living in the community and those residing in nursing homes. A study comparing the TUG results between elderly individuals living in the community versus those in nursing homes can help to understand differences in functional mobility between these two populations. The purpose of this study was to assess the reliability of the TUG in an elderly population. We also compared the scores of senior people living in the community to those in nursing homes in order to investigate dynamic balance scores and analyze potential discrepancies.

## 2. Materials and Methods

### 2.1. Participants

A cross-sectional study with two measurement points was performed as a mean to provide valuable information about the reliability of the TUG over time. By conducting reliability analysis research with two measurement points, researchers can evaluate the consistency and stability of the TUG and determine if the test results are reproducible. The study included a total of 117 participants, comprising 38 males and 79 females, with ages ranging from 60 to 92 years. The mean age of the participants was 77.19 ± 8.81 years, and the mean body mass index (BMI) was 28.04 ± 4.11 kg/m^2^. The distribution of BMI in the sample was as follows: normal weight (*n* = 21, 17.9%), overweight (*n* = 66, 56.4%), and obese (*n* = 30, 25.7%). Regarding subsamples, the study included a total of 49 community-living older adults (*n* = 38 females and 11 males), with ages ranging from 58 to 86 years (M = 74.38 ± 6.33). The study also included a total of 68 nursing home resident older adults (*n* = 41 females and 27 males), with ages ranging from 56 to 96 years (M = 79.21 ± 9.78).

Participants in this study were older persons who lived in the community or in nursing homes and met specified inclusion criteria. Participants were eligible if they were 60 or older. If a participant had a known diagnosis of a cognitive disorder or another neurological condition that could impair physical functioning, mobility, or balance, they were excluded. Participants were excluded from participation if they had a history of recent falls, defined as falling twice or more in the previous six months. Participants also had to be able to able to provide informed consent.

### 2.2. Data Collection Procedures

The data collecting procedures were approved by the Institutional Review Board and adhered to all Helsinki Declaration ethical criteria for human subjects research [17]. To establish a representative sample of the senior population, data for this study were collected in both nursing facilities and community settings. A convenience sampling method was used for data collection, because the researchers could have access to potential participants in both nursing facilities and community settings. Specifically, researchers contacted local nursing homes to obtain approval to conduct the current study. The study was carried out at nursing homes with the help of nursing home employees, who assisted in identifying potential participants based on the inclusion criteria. The researchers then briefed the participants about the goal of the study and received written informed consent. To maintain confidentiality, all data was taken in a private place. In the community, the researchers recruited volunteers through community programs [15,16,18]. Participants who were interested in the study were given information about it and the opportunity to ask questions. They provided written informed permission if they met the inclusion criteria and consented to participate.

Before beginning the TUG exam, participants were told of its purpose and procedures, and they were given the opportunity to ask questions. Participants were told they could opt out of the exam at any point if they felt uncomfortable or unsafe. Two experienced researchers then gave participants a clear and extensive description of the TUG test and demonstrated how to do it. During the exam, participants were allowed to utilize assistive devices they typically use, such as canes or walkers. Participants completed a practice trial to ensure that they understood the instructions and were comfortable doing the test. The researchers thoroughly observed the participants during the test to assure their safety. The experiment was carried out in a clear, open location devoid of obstructions, and the researchers gave physical assistance if needed. Participants were told to complete the exam at a comfortable and safe pace, but feedback was provided before the trial to conduct the test as fast as possible. Participants were given time to rest and recover after the exam, and any concerns or challenges were addressed. The time taken to complete the test was recorded in seconds using a stopwatch (T1). After 16 weeks, the participants completed the same test in the same conditions (T2).

### 2.3. Statistical Analysis

Prior to conducting the study, a sample size calculation for Intraclass Correlation Coefficient (ICC) analysis was performed using the Arifin calculator [19] to determine the necessary sample size to achieve adequate statistical power. The minimum acceptable reliability was estimated to be 0.70, with a desired significance level of 0.05 and power of 0.95, the calculated minimum sample size was 19. To account for potential dropouts and missing data (10%), to conduct the study, a minimum of 22 participants would need to be recruited. Intraclass Correlation Coefficient (ICC) analysis was conducted to assess the degree of agreement or consistency among multiple measurements of the TUG. The ICC ranges from 0 to 1, with higher values indicating greater agreement or consistency between the measurements. ICC values can be interpreted using commonly-accepted guidelines, such as those proposed by several authors [20,21], where an ICC value of less than 0.40 is considered poor agreement, 0.40–0.59 is fair agreement, 0.60–0.74 is good agreement, and 0.75 or higher is excellent agreement.

Prior to conducting planned *t*-test analyses, sample size calculation was performed using the Soper calculator [22] to determine the necessary sample size to achieve adequate statistical power. The anticipated effect size was estimated to be 0.5 based on previous research in the field [20,21]. With a desired alpha level of 0.05 and power of 0.95, the calculated minimum sample size was 64. To account for potential dropouts and missing data, to conduct the study, a minimum of 75 participants would need to be recruited.

## 3. Results

The results of the ICC analysis are reported in Table 1. As it stands, the ICC for both groups displayed acceptable scores. We also calculated bivariate correlations for additional evidence. The results showed that the correlation between T1 and T2 for community-dwelling elderly was r = 0.97, and the same correlation for nursing home resident older adults was r = 0.82. Thus, the results support the reliability of the TUG among elderly from two different resident homes. Considering the results from the *t*-test analyses, as theoretically expected, community-dwelling elderly displayed significant better scores than nursing home resident older adults in the T1 (F = 51.46; *t* [1, 115] = −6.68; *p* < 0.05) and T2 (F = 36.04; *t* [1, 115] = −5.45; *p* < 0.05) moments.

## 4. Discussion

The purpose of the present study was to assess the reliability of the TUG in a Portuguese elderly population. We also compared the scores of senior people living in the community to those in nursing homes in order to investigate dynamic balance scores and analyze potential differences. The results showed that the TUG displayed acceptable scores of ICC both in community-dwelling and nursing home resident elderly. In addition, significant differences were found between these groups of older adults, showing that community-dwelling elderly show greater agility and balance capacity compared to those living in nursing homes.

In the current study, the reliability of the TUG test was similar or close to those reported in previously published studies [23,24,25]. Many studies have used the ICC to assess the reliability of the TUG test. Shumway-Cook et al. (2000), for example, reported an ICC value of 0.98 for the TUG test in a sample of American elderly. Similar ICC results are reported in Japanese elderly [26] and Swedish older adults [25]. This implies that the test is very reliable and that results acquired on several occasions are likely to be consistent. In fact, the results displayed in the validation study of the TUG test by Podsiadlo and Richardson [10] that revealed an ICC value of 0.98 for the TUG test, confirming the high reliability of this test.

Statistically significant differences were found between community-dwelling elderly and those living in nursing homes regarding the TUG test results. These results are in accordance with previous literature [9,13]. According to studies, community-dwelling older persons have faster TUG test results than nursing facility residents. Verghese et al. [27] discovered that community-dwelling older persons had a mean TUG test result of 9.20 s, while nursing facility residents had a mean TUG test result of 19.20 s, which are similar in the present study. TUG test results from community-dwelling elderly and nursing home resident elderly were compared in an Italian study, and nursing home resident elderly had considerably longer TUG test times than community-dwelling elderly [28]. Another Brazilian study revealed that nursing home residents had greater TUG test times than community-dwelling seniors [29].

This disparity in TUG test results between community-dwelling elderly and those living in nursing homes could be attributed to a variety of factors, including disparities in overall health, mobility, and functional independence. The elderly in residential care homes are often frail. They have several diagnoses, a high medicine intake, a lack of general strength, and are prone to illness and disease. This participation of multiple systems probably generates sources of unpredictability and can be linked to a condition of unstable impairment or frailty [28]. Frailty is a disease or syndrome resulting from a multisystem decline in reserve capacity to the extent that a number of physiological systems are close to, or have passed, the threshold of symptomatic clinical failure. Frailty is thought to be the underlying cause of unbalance [29]. It should be noted that the TUG test is only one measure of functional mobility; other tests may be more effective for assessing functional mobility in other populations. While interpreting TUG test findings, it is also crucial to consider individual variances in health state, mobility, and functional independence.

### Limitations and Agenda for Future Research

The current findings should be restricted to healthy Portuguese elderly living in the community or nursing homes who can walk at least 10 m with or without a walking aid. Additionally, the degree of plantar flexor tone was not quantified, which may have influenced the current findings. We also did not measure agility and balance using other validated measures (e.g., Berg Balance Scale; Fall Efficacy Scale) that could serve for concurrent validity analysis. Information about premedical chronic disease history and magnitude may influence results. Gender influence was not explored in this study because there were fewer male elderly.

## 5. Conclusions

The results of the present study showed that the TUG presented acceptable scores of ICC both in community-dwelling and nursing home resident elderly. In addition, significant differences were found between these groups of older adults, showing that community-dwelling elderly show greater agility and balance capacity compared to those living in nursing homes. It is a quick and easy test that assesses how long it takes an individual to get out of a chair, walk 2.44 m, turn around, walk back to the chair, and sit down. The TUG test is a low-cost instrument for assessing functional ability in the elderly. It requires minimal equipment and can be carried out by a variety of healthcare professionals (e.g., exercise physiologists, nurses), reducing the requirement for specialist equipment or personnel.

## Figures and Tables

**Table 1:** Means, standard deviations and inter-class correlation coefficients.

Community-Dwelling Elderly (*n* = 49)			Nursing Home Resident Elderly (*n* = 68)		
T1	T2	ICC	T1	T2	ICC
4.65 (0.81)	4.48 (0.84)	0.97	18.90 (14.89)	16.11 (14.89)	0.82

Notes: Data in T1 and T2 are reported in Mean and Standard Deviation; brackets = standard deviation; ICC = Intra-Class Correlation coefficients.

## Data Availability

Data are available upon request from the contact author.
